# Lower Trapezius Transfer Using the Retrograde Keyhole Technique With an Achilles Tendon–Bone Allograft

**DOI:** 10.1016/j.eats.2025.103550

**Published:** 2025-04-26

**Authors:** Hyung-gyu Cho, Jeong-woo Kim

**Affiliations:** Department of Orthopedic Surgery, Wonkwang University School of Medicine, Iksan, Republic of Korea

## Abstract

For patients with irreparable rotator cuff tears and no arthritis progression, various treatment options can be considered, one of which is tendon transfer. In particular, latissimus dorsi transfer has conventionally been used for posterior-superior irreparable rotator cuff tears and is regarded as the standard procedure. Since lower trapezius transfer was first introduced in 2009, it has resulted in significant improvements in pain relief, range of motion, and functional scores. However, various complications have also been reported, with graft failure occurring in approximately 20% of cases, predominantly at the greater tuberosity. To address these issues, we describe a lower trapezius transfer using the retrograde keyhole technique with an Achilles tendon–bone allograft. This technique involves creating a keyhole along the biceps groove to insert the allograft bone plug and promote bone-to-bone healing for enhanced fixation strength, which is expected to reduce failure rates. To date, short-term follow-up results have shown resolution of the external rotation lag sign and improvements in range of motion, suggesting promising outcomes.

Lower trapezius transfer (LTT) was first introduced by Elhassan et al.^1^ in 2009, and it was reported that 32 of 33 patients showed significant improvements in pain, range of motion, and scores.^2^ Since then, various techniques have been introduced and arthroscopically assisted techniques have also been developed. Comparative studies with previously used latissimus dorsi transfer (LDT) have been performed extensively, with biomechanical and clinical studies reporting superior outcomes with LTT.^3^^,^^4^

However, a recent systematic review of LTT reported various complications,^5^ with graft failure (tear) rates reaching up to 19.4%. Baek et al.^6^^,^^7^ observed graft tears in 19.4% of primary LTT cases (7 of 36) and 75% of revision LTT cases (3 of 4), all of which occurred in the greater tuberosity (GT) of the allograft area. Therefore, we hypothesized that graft failure in LTT would be more likely to occur at the GT of the allograft area than at the lower trapezius tendon of the allograft area. We further assumed that achieving greater allograft fixation strength at the GT would be critical for improving graft survival rates. Accordingly, we describe a technique for LTT using an Achilles tendon–bone allograft in patients with posterior-superior irreparable rotator cuff tears (IRCTs) (Video 1).

## Surgical Technique

### Operative Indications

Patients are selected based on the following criteria: magnetic resonance imaging findings of an irreparable posterior-superior rotator cuff tear involving the infraspinatus, advanced fatty infiltration of the infraspinatus on sagittal oblique imaging (Goutallier grade 3 or 4),^8^ and positive findings of the external rotation lag sign on physical examination. Patients are excluded if the subscapularis shows a large full-thickness tear or fatty infiltration of grade 3 or higher, if glenohumeral arthritis is classified as Hamada stage 2 or higher,^9^ or if bone quality is poor. Irreparable tears are confirmed through diagnostic arthroscopy by observing whether the retracted tendon can be mobilized to the footprint; if not, the patient is excluded.

### Patient Positioning and Anesthesia

**Fig 1:**
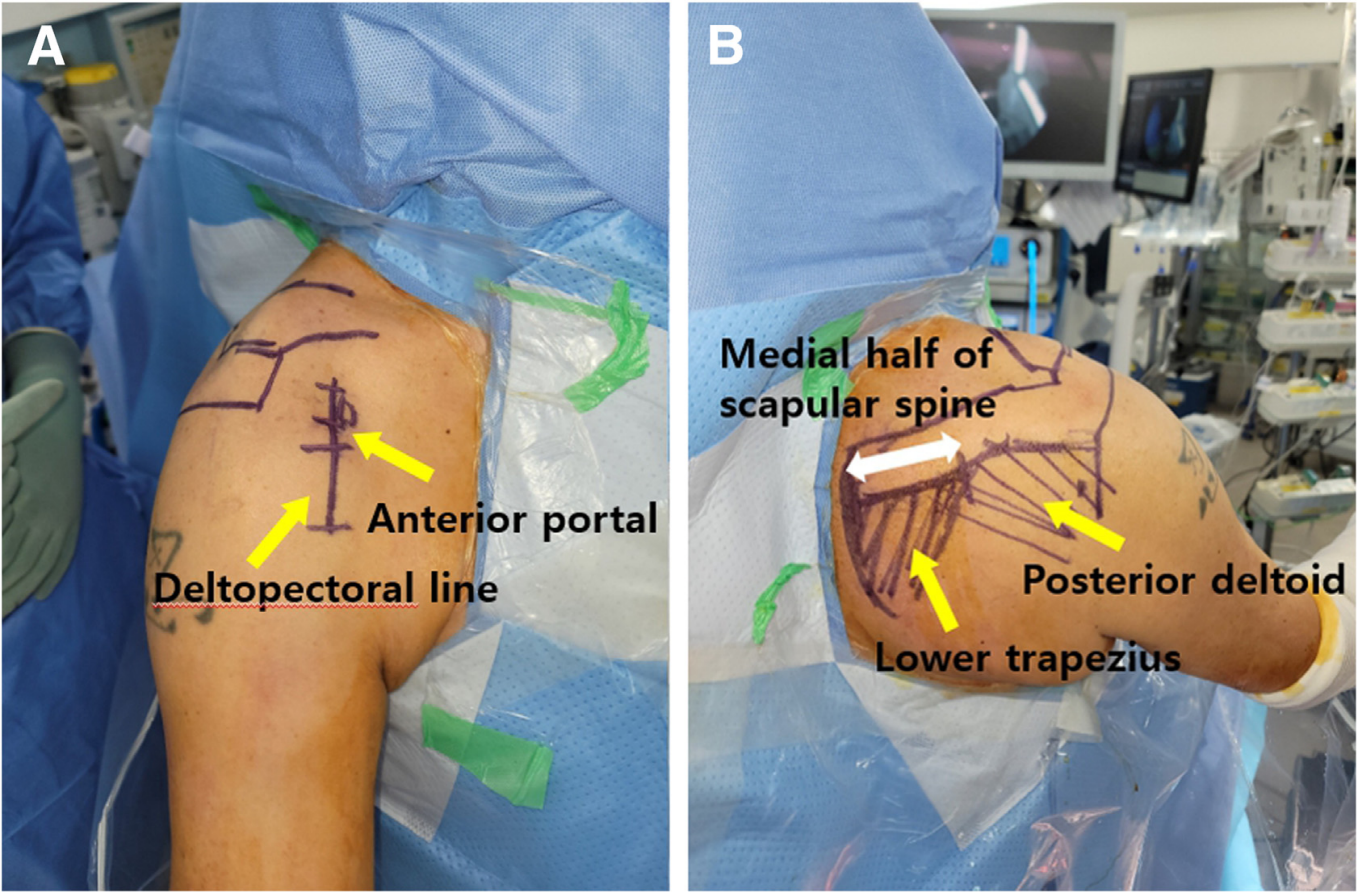
Beach-chair positioning (right shoulder). By use of a marking pen, the deltopectoral line is marked along the anterior portal, and the directions of the muscle fibers are used to indicate the lower trapezius and posterior deltoid. (A) Anterior aspect. (B) Posterior aspect.

Preoperatively, a peripheral nerve block and general anesthesia are administered. The patient is seated at 60° in the beach chair position, and both legs are elevated to maintain this position. Routine povidone-iodine skin preparation and standard surgical draping are performed. The shoulder joint is palpated with a marking pen to mark the anterior, lateral, and posterior borders of the distal end of the clavicle, acromion, acromioclavicular joint, scapular spine, and medial scapular border. The deltopectoral line is marked for grafting the Achilles tendon–bone allograft onto the biceps groove using the retrograde keyhole technique (Fig 1).

### Portal Placement and Diagnostic Arthroscopy

To form the posterior portal, a blunt trocar is placed 1 cm medial and 1 cm inferior to the posterolateral tip of the acromion and inserted into the glenohumeral joint, with a bladder trocar facing the coracoid process between the infraspinatus and teres minor tendons. An anterior spinal needle is placed on the soft-tissue spot in a slightly lateral portion (to suit the deltopectoral incision) of the coracoid process to the anterior portal.

If the biceps tendon is still present in the joint (often absent owing to a chronic tear of the long head), it is examined for signs of impingement, such as fraying, partial tear, or inflammatory changes. If the tendon is well margined and intact, it can be rerouted for augmentation to support supraspinatus repair or posterior marginal convergence. Otherwise, it is removed from the superior glenoid. Any loose bodies are excised, and synovectomy is performed as necessary.

Subsequently, the scope (ConMed Linvatec, Largo, FL) is repositioned in the subacromial space. A lateral portal is established, typically anterolaterally and posterolaterally. Bursectomy is performed, followed by a thorough evaluation, characterization, and mobilization of the rotator cuff tear to confirm that it is neither irreparable nor advisable to repair. LTT is considered in cases of massive, irreparable, full-thickness tears of the supraspinatus and infraspinatus, provided that there is no severe degenerative change in the glenohumeral joint. After the decision to perform LTT is made, the biceps groove is exposed through the deltopectoral approach in the intraoperative field, and the Achilles tendon–bone allograft is prepared on the back table.

### Lower Trapezius Tendon Harvest

**Fig 2:**
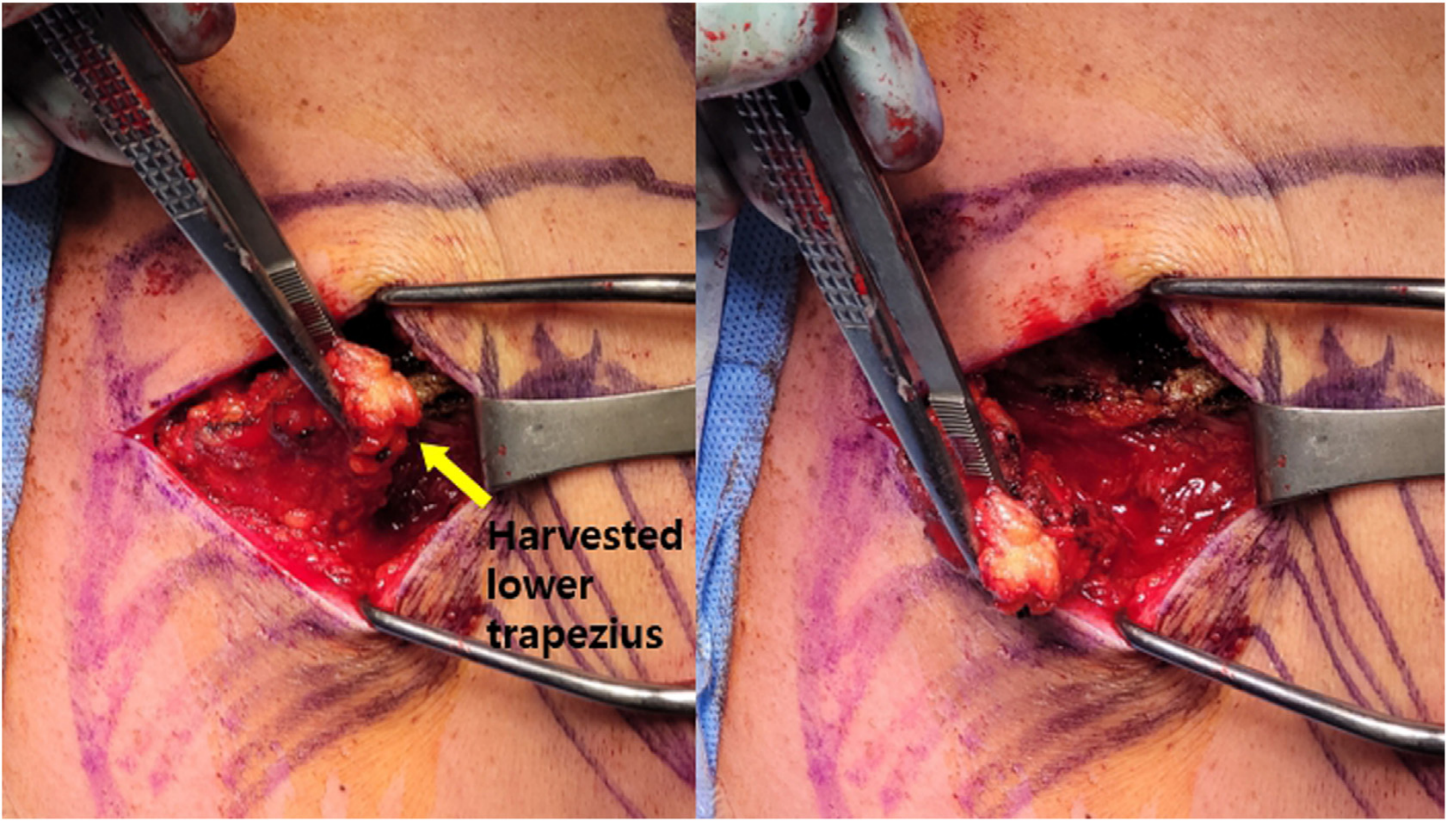
Lower trapezius harvest (right shoulder). The lower trapezius is harvested along the footprint on the medial half of the scapular spine, and sufficient medial release is performed for mobilization.

The locations of the scapular spine and medial border of the scapula are identified and marked, and a longitudinal incision is made along the inferior border of the scapular spine, encompassing the medial half. The skin and subcutaneous tissue are reflected, revealing obliquely oriented muscle fibers running in an inferomedial-to-superolateral direction. Meticulous periosteal peel off is performed at the footprint of the scapular spine. Medial release of the lower trapezius is performed to ensure mobilization with careful attention paid to the accessory spinal nerve (Fig 2).

### Preparation of Biceps Tendon and Biceps Groove

**Fig 3:**
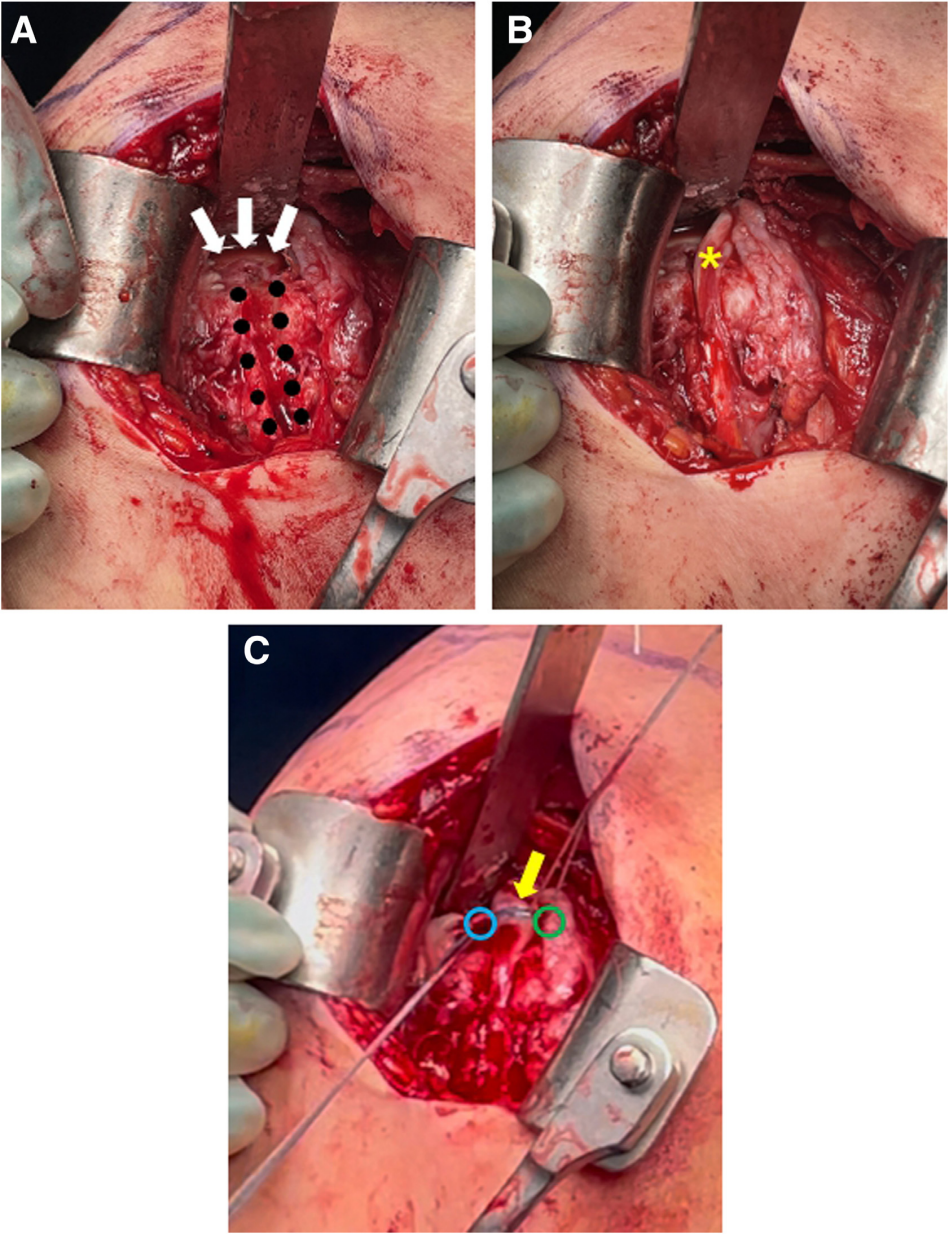
Biceps rerouting (right shoulder). (A) Biceps rerouting is performed by deepening the greater tuberosity using a burr or microsaw along the supraspinatus line in the supraspinatus footprint area. The white arrows indicate the humeral head, and the black dots indicate the newly formed biceps groove. (B) The biceps tendon (yellow asterisk) is repositioned. (C) The biceps tendon is secured using suture compression (yellow arrow). The green circle indicates the medial anchor, and the blue circle indicates the lateral knotless anchor.

An additional 5-cm skin incision is made through the deltopectoral approach in the anterior portal. The humeral head is exposed using a Kölbel self-retractor. If a biceps tenotomy has been performed previously, the biceps tendon is detached from the biceps groove and tagged with No. 2 Ethibond sutures (Ethicon, Somerville, NJ) for subsequent tenodesis. However, if the biceps tendon is well margined and intact, biceps rerouting is performed to expose the biceps groove. Biceps rerouting is performed by deepening the GT using a burr or microsaw along the supraspinatus line in the supraspinatus footprint area, similar to its use as a structural scaffold in superior capsular reconstruction (Fig 3). The biceps tendon is then repositioned and secured. Biceps fixation is performed at 60° of abduction and 60° of external rotation. A medial anchor with 6 threads (4.8-mm Healmax Medial Anchor; Osteonic, Seoul, Republic of Korea) is inserted on the anterior side of the newly formed biceps groove. A lasso-loop suture is applied using 2 threads at the point where the biceps tendon achieves appropriate tension, whereas the other 2 threads are used for suture compression with a lateral knotless anchor (3.5-mm Pop-Lock; ConMed Linvatec) placed directly posterior to the newly formed biceps groove. The remaining 2 threads are reserved for later use in suture compression of the Achilles tendon–bone graft onto the GT footprint.

Decortication is performed along the exposed biceps groove. A tunneling guide pin (ACL Drill Guide, AR-1875; Arthrex, Naples, FL) used for anterior cruciate ligament reconstruction is inserted vertically, 5 mm posterior to the cortex of the biceps groove, from proximal to distal. A 10-mm reamer (Cannulated Headed Reamer, AR-1410; Arthrex) is used to create a tunnel for tendon-bone insertion, ensuring a minimum depth of 30 mm to accommodate the 30-mm length of the allograft bone plug. The procedure aims to remove only the inner cortex of the biceps groove, located between the ridge formed by the lesser and greater trochanters. For any incompletely removed areas, a small osteotome is used to fully remove the inner cortex. Therefore, caution is exercised to avoid removing the lateral aspect of the lesser trochanter and the medial aspect of the greater trochanter during the procedure.

### Graft Preparation

**Fig 4:**
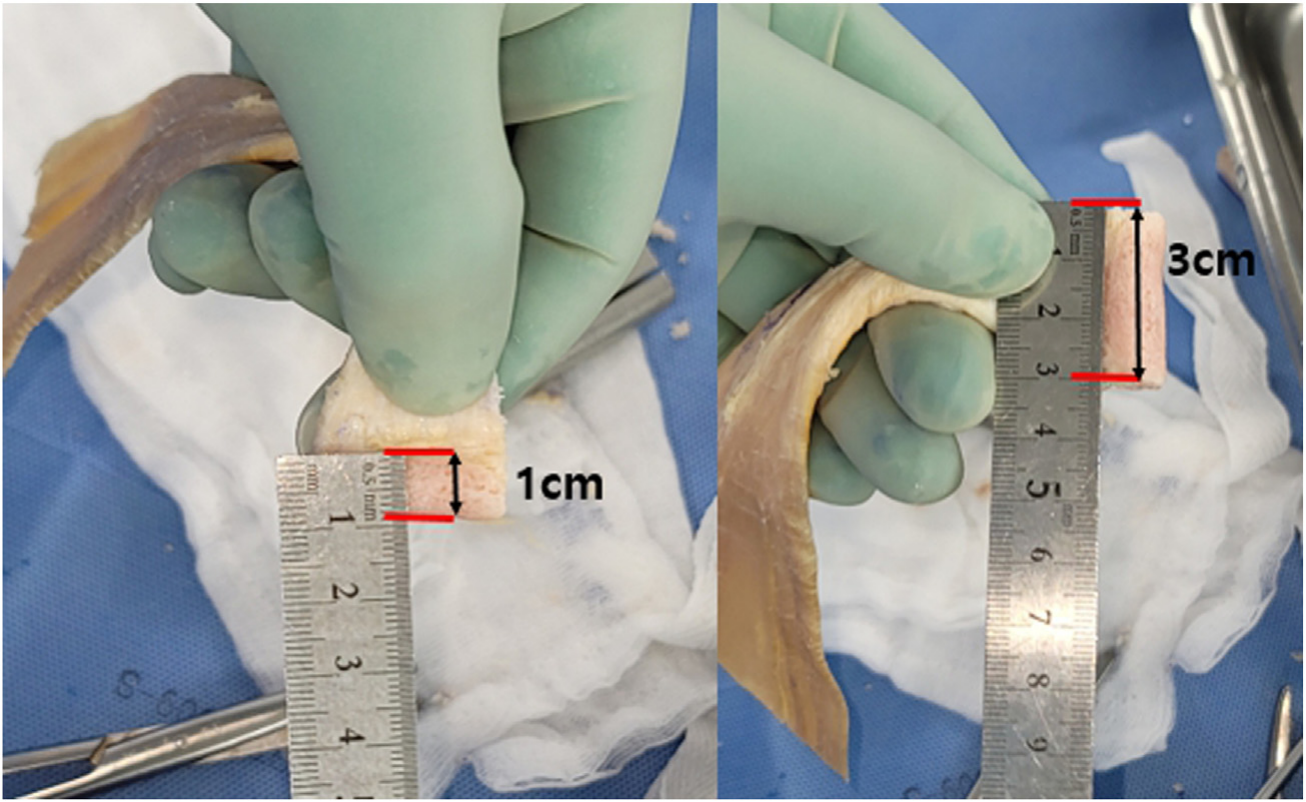
Graft preparation: Achilles tendon–bone graft with bone plug measuring 10 mm in diameter and 30 mm in length.

An Achilles tendon–bone allograft is prepared on the back table. After the allograft is thawed, a burr, microsaw, rasp, and rongeur are used to make a 10-mm transverse bone plug with a 30-mm length from the calcaneus along with the attached tendon (Fig 4). The Achilles tendon has a sufficient length of approximately 18 to 20 cm to allow tendon-to-tendon suturing of the lower trapezius tendon. Because the tendon becomes thinner and wider as it extends away from the bone, approximately 5 cm of the distal end is folded horizontally into 2 layers and sutured in a Krackow fashion using No. 2 Ethibond sutures to facilitate its passage through the tunnel and infraspinatus fascia.

### Graft Fixation Along Biceps Groove Using Keyhole Technique

**Fig 5:**
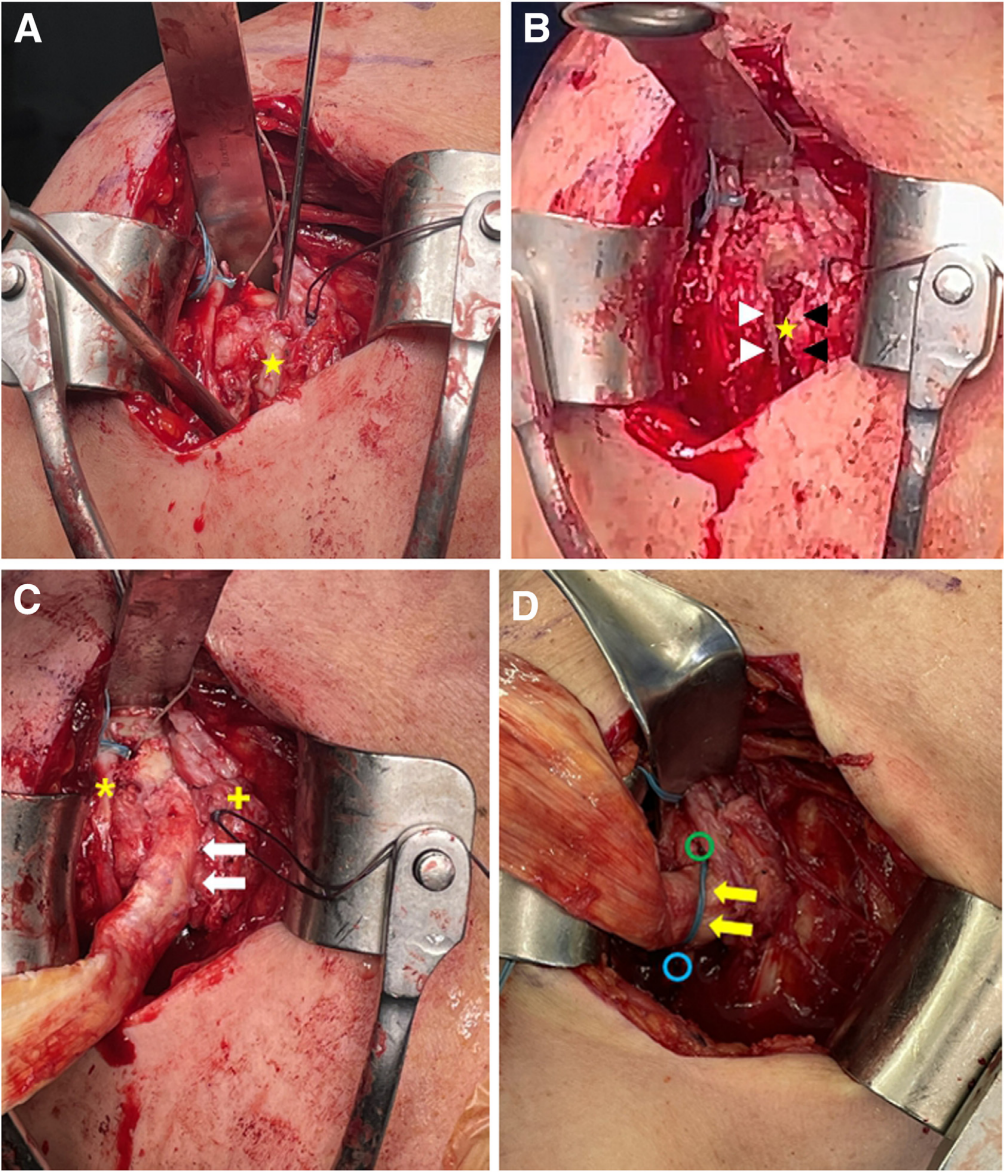
Graft insertion and fixation (right shoulder). (A, B) A tunneling guide pin is inserted vertically, 5 mm posterior to the cortex of the biceps groove (yellow stars), proximal to distal. A 10-mm reamer is used to create a tunnel for tendon-bone insertion. The black and white arrowheads indicate the ridge formed by the lesser and greater trochanters. (C, D) The Achilles tendon–bone graft is inserted into the humeral hole and fixed using suture compression (yellow arrows). The white arrows indicate the inserted graft; yellow asterisk, re-routed biceps tendon; yellow cross, subscapularis; green circle, medial anchor; and blue circle, lateral knotless anchor.

The Achilles tendon–bone graft is inserted vertically into the humeral hole along the biceps groove using a bone tamper. After the bone plug is fully inserted, the Achilles allograft is pulled to ensure secure fixation in the biceps groove and to confirm the presence of a hinged action. A medial anchor (Y-Knot RC All-Suture Anchor with 2 No. 2 Hi-Fi Sutures; ConMed Linvatec) with 4 threads is inserted just lateral to the biceps groove and just proximal to the allograft tendon. Two threads are used for suture compression using a lateral knotless anchor (3.5-mm Pop-Lock) placed directly distal to the allograft tendon by wrapping the tendon with sutures and pressing it down from top to bottom, whereas the remaining 2 threads are used for repair if a repairable subscapularis tear is present. Soft-tissue or anchor-based tenodesis is performed in the area below the keyhole for the previously tenotomized biceps tendon (Fig 5).

### Arthroscopically Guided Graft Passage

An arthroscope is inserted through the anterolateral portal and advanced along the infraspinatus to create a graft passage through the infraspinatus fascia beneath the posterior deltoid. The graft passage location is identified, and a grasper is inserted from outside to inside at the target location. The No. 2 Ethibond sutures attached to the allograft tendon are then pulled through to complete the graft passage.

### Additional Suture Compression on GT Footprint

**Fig 6:**
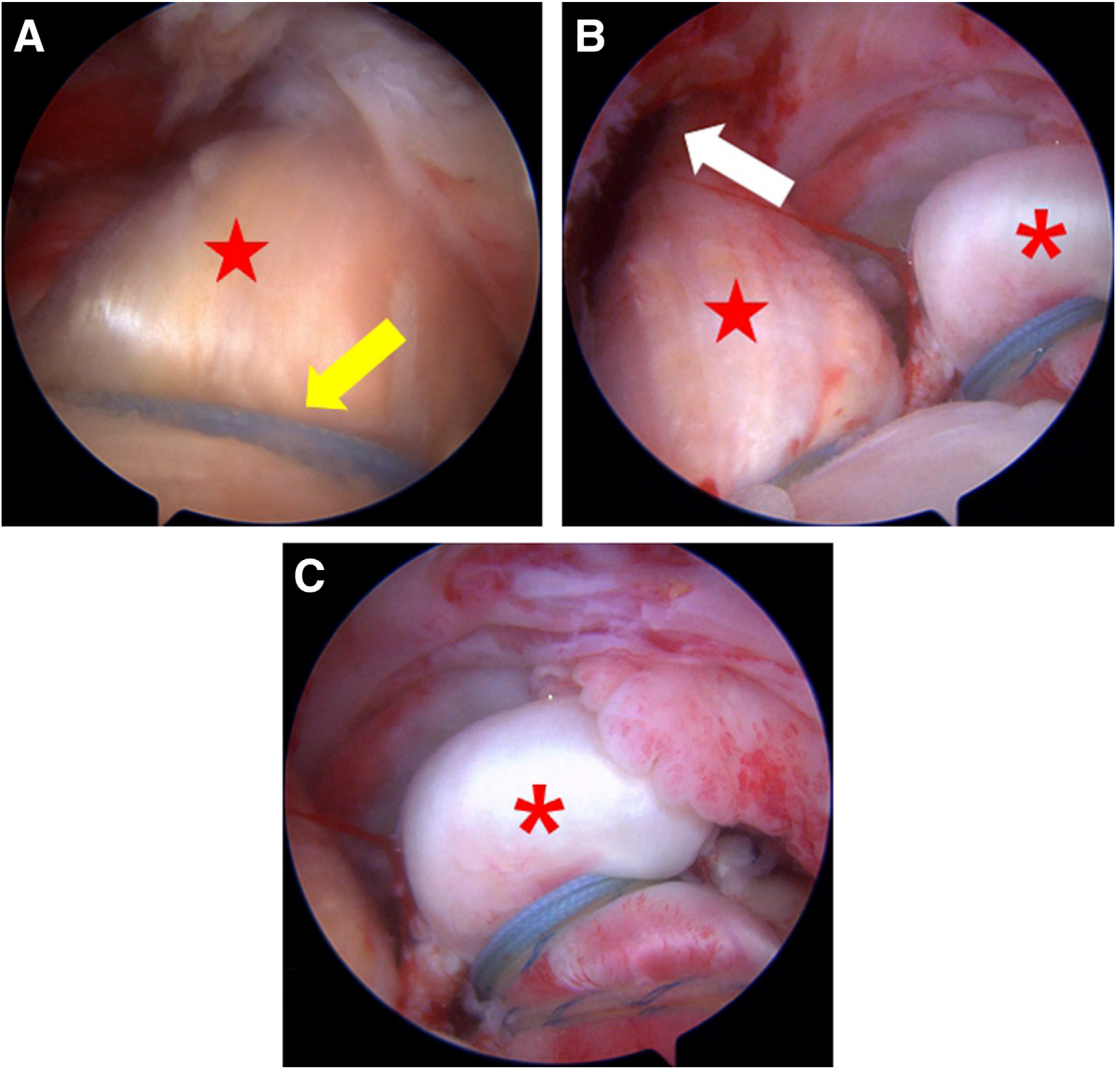
Securing of graft (red stars) on greater tuberosity footprint (right shoulder, beach-chair position). (A) The Achilles tendon–bone graft is secured onto the greater tuberosity footprint (with posterolateral portal as viewing portal). (B, C) The graft passes through the infraspinatus fascia in alignment with the direction of the infraspinatus (with anterolateral portal as viewing portal). The red asterisks indicate the biceps tendon; yellow arrow, suture compression; and white arrow, graft passage.

Anatomically, the normal supraspinatus and infraspinatus muscles are attached to and act on the GT. In this procedure, securing the Achilles tendon–bone graft onto the GT footprint ensures that the transferred lower trapezius applies force to the humerus in a manner similar to that of the infraspinatus. The position and orientation of the graft in the subacromial space are evaluated under arthroscopic guidance. If deemed appropriate, additional suture compression is performed using the remaining 2 threads from the medial anchor used for biceps fixation. If a biceps tenotomy has been previously performed, the medial anchor (Y-Knot RC All-Suture Anchor with 2 No. 2 Hi-Fi Sutures) is placed directly medial to the allograft tendon. Two threads are used for suture compression using a lateral anchor (4.5-mm Reelx STT Knotless Anchor; Stryker, Kalamazoo, MI) placed directly lateral to the allograft tendon by wrapping the tendon with sutures and pressing it down from top to bottom (Fig 6).

### Suturing of Allograft Tendon to Lower Trapezius Tendon

**Fig 7:**
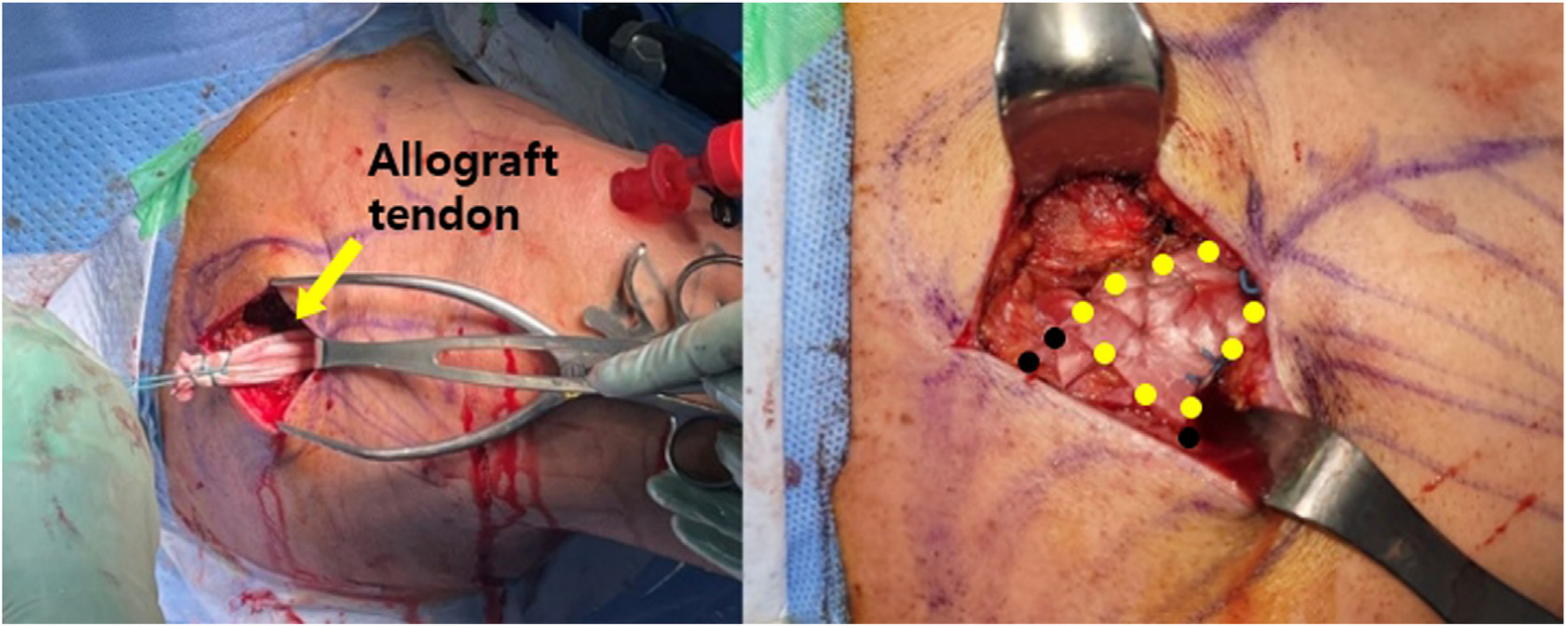
Suturing of allograft tendon (yellow dots) to lower trapezius tendon (right shoulder). The allograft is sutured to the lower trapezius tendon using fish-mouth and Krackow sutures. The black dots indicate the harvested lower trapezius.

Suturing between the lower trapezius and allograft tendon is performed at 60° of external rotation and 60° of abduction. After tension is applied to the allograft to identify the appropriate location for window formation, a longitudinal incision is made in the allograft and a fish-mouth suture is performed using No. 2 Ethibond sutures. Because the allograft tendon is relatively wider and thinner than the lower trapezius, it is wrapped around the lower trapezius and secured using the Krackow suture technique with No. 2 Ethibond sutures (Fig 7). After suturing is completed, the fixation strength is assessed to ensure that it is adequate. Under arthroscopic guidance, it is confirmed that the graft passes smoothly through the passage during rotation.

### Closure and Postoperative Protocol

All the portals are closed in a standard manner. Postoperatively, the patient is placed in a sling using an abduction brace. The brace should remain in place for 8 weeks. Passive external rotation exercises are allowed immediately after surgery and should be performed to the maximum extent possible within a pain-free range. Other progressive motions such as forward elevation are passively initiated at 4 weeks postoperatively. Full active motion is allowed after 8 weeks, and strengthening exercises progress after 12 weeks. Return to activities requiring overhead lifting is allowed no earlier than 16 weeks. A full return to activities is allowed at 6 months postoperatively.

## Discussion

We describe the LTT technique using an Achilles tendon–bone graft. Tendon transfer is a well-established option for reconstructing the lost function of a posterior-superior IRCT, particularly in younger or more active patients without significant degenerative changes. Recently, LTT has emerged as an alternative option for restoring shoulder external rotation. Since it was first described by Elhassan et al.,^1^ several clinical studies have confirmed the effectiveness of this transfer in improving shoulder external rotation.

Elhassan et al.^2^ performed LTT in 33 patients with posterior-superior IRCTs. At a mean 47-month follow-up, significant improvements in pain and range of motion, including forward flexion, abduction, and external rotation, were observed. In several studies comparing LTT and LDT, LTT has shown superior outcomes. A biomechanical study revealed that the lower trapezius, compared with the latissimus dorsi, generated a greater abduction moment when transferred to the supraspinatus and infraspinatus insertion sites.^3^ In addition, both the lower trapezius and latissimus dorsi showed strong external rotation moment arms at the infraspinatus insertion site. A clinical study further showed that although both LDT and LTT improved the overall clinical outcomes of patients with posterior-superior IRCTs, LTT exhibited superior outcomes in terms of shoulder range of motion, functional improvements, and progression of arthritis.^4^

One complication of LTT is graft failure (tear), with reported rates reaching up to 19.4%.^6^^,^^10^^,^^11^ Few studies have addressed the specific site of graft tear; however, Baek et al.^6^ reported that among 36 patients, 7 experienced graft tears, all of which occurred at the GT to the allograft area, instead of at the lower trapezius tendon to the allograft area. In addition, Baek et al.^7^ performed revision LTT in 4 cases of failed LTT and observed graft retears in 75% of these cases (3 of 4), all of which occurred at the GT. Therefore, to reduce graft tears occurring at the GT, we introduce the keyhole technique to promote bone-to-bone healing (Tables 1 and 2).

**Table 1:** Technical Pearls and Pitfalls of Lower Trapezius Transfer Using Achilles Tendon–Bone Allograft

During the lower trapezius tendon harvest, including the triangular fat tissue helps achieve an appropriate thickness.
During biceps rerouting, tenodesis should be performed with sufficient tension applied to the position of abduction and external rotation. This helps avoid kinking within the intra-articular space.
The graft diameter should measure 10 mm via the graft sizing block.
When the surgeon is forming the keyhole, the tunneling guide pin should not be placed within 5 mm posterior to the inner cortex to prevent ridge removal and fracture.
For graft suture compression using knotless anchor fixation, gentle handling should be applied because the fixation site involves the diaphysis, which may lead to fractures. Drilling can be performed if necessary.
The graft should not flip or twist within the intra-articular space during passage.
Postoperatively, passive external rotation exercises are initiated immediately and should be performed to the maximum extent possible within a pain-free range.

**Table 2:** Advantages and Disadvantages of Surgical Technique

Advantages
Bone-to-bone fixation provides stronger stability than tendon-to-bone fixation.
A subscapularis tear can be repaired if present.
Rerouting the biceps tendon to the greater tuberosity provides an additional stabilizing effect similar to superior capsular reconstruction.
Disadvantages
A mini-open incision is required for humeral head exposure, instead of an all-arthroscopic procedure.
Improper positioning during keyhole formation or anchor fixation may lead to fracture.
Use of an allograft incurs additional costs.

In conclusion, LTT using the retrograde keyhole technique with an Achilles tendon–bone allograft may yield favorable outcomes when applied to appropriately selected patients. However, larger studies with greater numbers of patients and long-term follow-up are required to comprehensively evaluate its effectiveness.

## Disclosures

The authors declare the following financial interests/personal relationships which may be considered as potential competing interests: H-g.C. reports that this research was supported by a grant from 10.13039/501100002569Wonkwang University (2024). J-w.K. reports that this research was supported by a grant from Wonkwang University (2024).

## Supplementary Data

Video 1 Surgical technique. Arthroscopic findings show a posterior-superior irreparable rotator cuff tear. The condition of the biceps tendon is also evaluated. The lower trapezius tendon is harvested first. The lower trapezius tendon is detached from its footprint, and medial release is performed bluntly. Through the deltopectoral approach, the humeral head is exposed. The greater tuberosity is deepened, and biceps rerouting is performed, serving as a structural scaffold similar to superior capsular reconstruction. Suture compression is additionally applied using anchors. A guide pin is placed parallel to and 5 mm posterior to the biceps groove, and a 10-mm reamer is used to create a keyhole. A small osteotome and burr are used to completely remove only the inner cortex. An Achilles tendon–bone allograft with a 10-mm transverse bone plug and a length of 30 mm is prepared and inserted into the keyhole. The graft’s hinge action is checked, and suture compression is applied using anchors. If a subscapularis tear is present, the remaining threads are used for repair. Arthroscopic findings confirm the appearance of the rerouted biceps tendon. Under arthroscopic guidance, the graft passage is verified, and a grasper is inserted from outside to inside at the target location. The sutures attached to the allograft tendon are pulled through to complete the graft passage. Additional suture compression is applied to the greater tuberosity footprint. In the final step, suturing is performed between the allograft tendon and the lower trapezius tendon in 2 stages. After suturing is completed, the fixation strength is assessed to ensure it is adequate.
